# Coenzyme Q10 Activates the Antioxidant Machinery and Inhibits the Inflammatory and Apoptotic Cascades Against Lead Acetate-Induced Renal Injury in Rats

**DOI:** 10.3389/fphys.2020.00064

**Published:** 2020-02-07

**Authors:** Wafa A. AL-Megrin, Doaa Soliman, Rami B. Kassab, Dina M. Metwally, Ahmed E. Abdel Moneim, Manal F. El-Khadragy

**Affiliations:** ^1^Department of Biology, Faculty of Science, Princess Nourah Bint Abdulrahman University, Riyadh, Saudi Arabia; ^2^Department of Zoology and Entomology, Faculty of Science, Helwan University, Cairo, Egypt; ^3^Department of Parasitology, Faculty of Veterinary Medicine, Zagazig University, Zagazig, Egypt; ^4^Department of Zoology, Faculty of Sciences, King Saud University, Riyadh, Saudi Arabia

**Keywords:** coenzyme Q10, lead acetate, kidney, oxidative stress, inflammation, apoptosis

## Abstract

The kidney is among the metabolic organs most susceptible to injury, particularly following exposure to xenobiotics and heavy metals. We aimed to explore the potential protective impacts of coenzyme Q10 (CoQ10) on lead acetate (PbAc)-induced nephrotoxicity in rats. Four experimental groups (*n* = 7) were applied as follows: control group, CoQ10 alone (10 mg/kg), PbAc alone (20 mg/kg), and PbAc with CoQ10. Exposure to PbAc led to the accumulation of Pb in the kidney and increased urea and creatinine serum levels. The deposition of Pb coupled with the elevation of malondialdehyde and nitrate/nitrite levels along with the upregulation of inducible nitric oxide synthase. Additionally, upon PbAc poisoning, glutathione content and the antioxidant enzymes were depleted along with the downregulation of Nrf2 and HO-1 expression. Moreover, PbAc injection increased the protein and mRNA levels of pro-inflammatory cytokines namely, tumor necrosis factor-alpha and interleukin-1 beta, while decreased the levels of interleukin-10, an anti-inflammatory cytokine, in the kidney. Furthermore, exposure to PbAc correlated with increased levels of pro-apoptotic markers, Bax and caspase-3, and reduced levels of the anti-apoptotic marker Bcl-2. The administration of CoQ10 alleviated the molecular, biochemical and histological changes following PbAc intoxication. Thus, CoQ10 reduces the deleterious cellular side effects of PbAc exposure due to its antioxidant, anti-inflammatory and anti-apoptotic effects.

## Introduction

Lead (Pb) is a naturally occurring heavy metal in the earth’s crust; its widespread extraction and excessive use in anthropogenic activities have contaminated the environment and increased human exposure rate, resulting in the development of significant public health problems worldwide (Wani et al., 2015). Pb founds in three different states: organolead, such as tetramethyl lead (which is the most toxic form), metallic lead and inorganic lead (di- and tetravalent salts) (Patrick, 2006). Pb is used in the production of batteries, lead-based alloys and metal products, such as sheet lead, solder and pipes, and in, ammunition, cable covering, and other products. The primary pathways of human exposure to Pb are inhalation and ingestion (Wani et al., 2015). Independently of the route of bio-uptake, the absorbed Pb is primarily eliminated from the body through urine and feces; whereas, sweat, saliva, skin, breast milk, and seminal fluids are minor routes of Pb-excretion (Sears et al., 2012). Pb toxicity is an important environmental disease and its effects on the human body are devastating. Pb exposure causes serious health hazards in animals and humans including various forms of cancer, gastrointestinal problems, hepatotoxicity, nephrotoxicity, central nervous system impairments and cardiovascular diseases (Korsrud and Meldrum, 1988; Abdel Moneim, 2016; Dkhil et al., 2016; Sy et al., 2019).

Pb accumulates in high concentration mainly in the bones, but the kidney is the primary starting site for its accumulation (Orr and Bridges, 2017). Duration and magnitude of exposure, as well age and genetics are important and critical factors determining the toxic effects and symptoms of Pb (Garcia-Esquinas et al., 2015). Chronic exposure to Pb resulted in Pb accumulation in the proximal tubules, which leads to injury and eventually to kidney failure. Pb toxicity can also lead to Fanconi syndrome, characterized by polyuria, loss of amino acid, glucose, protein, calcium, and phosphate in urine (Garcia-Esquinas et al., 2015). Acute Pb toxicity causes injury of the proximal tubular structure and many histological alterations including mitochondrial swelling and rupture, and eosinophilic intranuclear inclusions in tubular cells consisting of Pb-protein complexes (Orr and Bridges, 2017). The kidney injury may be revealed by elevated urine urate, vasoconstriction, and following by glomerulosclerosis, hypertension and interstitial fibrosis (Garcia-Esquinas et al., 2015).

Coenzyme Q10 (CoQ10 or ubiquinone-10) is a fat-soluble compound composed of a benzoquinone ring with 10 isoprene side-chains (Awad et al., 2018). It is synthesized naturally by the human body utilizing a group of enzymes (CoQ complexes) situated in the mitochondrial matrix intermembrane (Awad et al., 2018). Along the inner membrane of the mitochondrion, CoQ_10_ plays a pivotal role in the aerobic cellular respiration for the production of ATP, where the electron transport chain utilizes it as an electron acceptor in oxidative phosphorylation, transforming products of metabolism into ATP (Lee et al., 2017). CoQ10 is ubiquitously expressed in most human tissues with the highest concentrations found in the liver, heart, and kidney (Potgieter et al., 2013). CoQ10 is widely found in many animals used as protein sources like beef, chicken, lamb, pork, and fish. It is also found in vegetables like broccoli, spinach, soybean, palm oils, canola, nuts, and legume, and fruits like strawberry, orange, and apple (Bhagavan and Chopra, 2006; Parmar et al., 2015).

CoQ10 has been shown to protect against cancer, neurodegenerative disorders, heart disease, kidney injury, and diabetes (Turunen et al., 2004; Lance et al., 2012). CoQ10 also acts as a soluble antioxidant free radical quencher that minimizes the production and propagation of reactive oxygen species (ROS) that cause oxidative stress owing to their detrimental effects on DNA, proteins, lipids, and the overall mitochondria dysfunction (Lance et al., 2012; Sohet and Delzenne, 2012). CoQ10 stabilizes the cell membrane and the intracellular membranes by protecting the phospholipids of the membranes from peroxidation (Sohet and Delzenne, 2012). In addition, CoQ10 can increase the production of key antioxidants (Blatt and Littarru, 2011; Belliere et al., 2012). Moreover, CoQ10 has been shown to have anti-apoptotic and inflammatory activities (Sy et al., 2019). Hence, the current investigation was undertaken to assess the protective effect of CoQ10 against Pb-induced kidney injury through examining the accumulation of Pb in the renal tissue, and its effects on kidney function markers, kidney relative weight, redox homeostasis, and inflammatory and apoptotic mediators in rats.

## Materials and Methods

### Chemicals and Experimental Animals

Pb (II) acetate trihydrate (Pb(CH_3_CO_2_)_2_.3H_2_O; CAS Number 6080-56-4) and CoQ10 (C_59_H_90_O_4_; CAS Number: 303-98-0) were sourced from Sigma-Aldrich (St. Louis, MO, United States). The remaining chemicals and reagents utilized in the present study were of high purity grade. CoQ10 was prepared in normal saline solution (0.9% NaCl) containing 1% Tween 80 (v:v) by mixing overnight at 25°C and kept in a dark bottle to avoid its decomposition.

A total of 28 adult male Wistar albino rats (10 weeks old, 150 ± 20 g) were sourced from the animal facility of VACSERA (Cairo, Egypt), and kept in wire polypropylene cages under typical laboratory environment (temperature 25 ± 2°C and artificial 12 h light/12 h dark cycle). The rats were fed with animal standard pellet diet and water *ad libitum* and allowed to acclimatize 1 week prior to the study. All experimental protocols were performed in according with the European Community Directive (86/609/EEC) and the study was approved by the Institutional Animal Ethics Committee guidelines for animal care and use of the Zoology Department at Helwan University (Approval Number: HU/Z/010-18).

### Dosage Selection

In the current study, lead acetate (PbAc) was injected i.p. (intraperitoneally) daily at a dose of 20 mg/kg bwt according to Abdel Moneim (2012). The selected dose is equivalent to 1/30 of the LD_50_ used to produce acute toxicity in rats. Moreover, this dose was found to induce renotoxicity as evidenced by the development of oxidative, inflammatory and apoptotic reactions. Meanwhile, the selected CoQ10 dose (10 mg/kg) was found previously to inhibit the pathological changes associated with renal impairments following sodium arsenite (Adil et al., 2015).

### Experimental Design

To explore the protective impacts of CoQ10 on PbAc-induced nephrotoxicity, the rats were separated into four groups: the control group, CoQ10 alone, PbAc alone, and PbAc with CoQ10 (each group containing seven rats). The control group was i.p. injected with 0.1 ml of saline containing 1% Tween 80 (v:v). CoQ10 was injected i.p. daily at a dose of 10 mg/kg at 10:00 AM to non-fasted rats according to Fouad and Jresat (2012), while PbAc was injected i.p. daily at a dose of 20 mg/kg following Abdel Moneim (2012). The CoQ10 was post-administered 1 h after the PbAc injection. All groups were i.p. injected for 7 days.

The rats were sacrificed by using an overdose of isoflurane 24 h after the last injection. Blood was taken from the abdominal aorta, and the serum sample was separated. The left kidney was excised carefully, weighed and immediately homogenized in ice-cold buffer of 50 mM Tris–HCl (pH 7.4) to prepare a 10% (w/v) homogenate. The supernatants were obtained by centrifugation of the homogenates at 3000 × *g* for 10 min at 4°C. The obtained supernatants were stored at −80°C for the various biochemical analyses, while the right kidney was kept for Pb concentration determination and histopathological examination.

### Lead Concentration in the Kidney Tissue

Pb content in the kidney tissue was estimated by flame atomic absorption spectrophotometer according to the method of Szkoda and Zmudzki (2005). The amount of Pb in the kidney was expressed as μg/g wet kidney tissue.

### Kidney Function Assays

Serological concentrations of serum urea and creatinine were measured using specific commercial kits according to manufacturers’ procedures.

### Oxidative Stress Markers in the Renal Tissue

Lipid peroxidation (LPO) was estimated by determining the concentration of malondialdehyde (MDA), an end product of lipid peroxidation, based on the method of Ohkawa et al. (1979). Nitric oxide (NO) content in the kidney homogenates was assessed according to Green et al. (1982). Whereas, the concentration of renal reduced glutathione (GSH) was determined by using 5,5′-dithiobis (2-nitrobenzoic acid) based on the described method of Ellman (1959).

### Antioxidant Status in the Renal Tissue

The activity of renal superoxide dismutase (SOD) was determined based on the reduction of the nitroblue tetrazolium (NBT) dye (Sun et al., 1988). Renal catalase (CAT) was estimated by mixing 50 μl of renal supernatant with 30 mM hydrogen peroxide in 50 mM of potassium phosphate buffer (pH 8.0), and the decomposition of hydrogen peroxide was measured at 240 nm for 2 min at 30 s intervals (Luck, 1965). Glutathione reductase (GR) activity was determined indirectly by measuring the oxidation of NADPH to NADP^+^ at 240 nm (Factor et al., 1998). Glutathione peroxidase (GPx) activity was assayed using the method of Paglia and Valentine (1967).

### Inflammation Marker Assays

Renal levels of pro-inflammatory cytokines namely, tumor necrosis factor-α (TNF-α) and interleukin-1β (IL-1β) and the anti-inflammatory cytokine, 10 (IL-10), were measured using kits sourced from Thermo Fisher Scientific according to the manufacturer’s procedures.

### Apoptotic Marker Assays

Renal levels of Bcl-2, Bax and caspases-3 were measured using kits [Bax (BioVision, Inc., Catalog # E4513) caspase-3 (Sigma-Aldrich, Catalog # CASP3C-1KT) and Bcl-2 (Cusabio, Catalog # CSB-E08854r)] according to the manufacturers’ protocols.

### Quantitative Real-Time PCR

Total RNA was extracted from the kidney specimens using a RNeasy Plus Minikit (Qiagen, Valencia, CA, United States). Then, complementary DNA (cDNA) was synthesized by using the RevertAid^TM^ H Minus Reverse Transcriptase (Fermentas, Thermo Fisher Scientific Inc.). The synthesized cDNA was analyzed in triplicate with real-time PCR. Real-time PCR was performed with Power SYBR^®^ Green (Life Technologies, Carlsbad, CA, United States) on an Applied Biosystems 7500 system. β-Actin (*Actb*) was used for the normalization of gene expression. Primer sequences of the examined genes are provided in Table 1.

**TABLE 1:** Primer sequences of genes analyzed in real-time PCR.

**Name**	**Sense (5′—3′)**	**Antisense (5′—3′)**
*Actb*	GCAGGAGTACGATGAGTCCG	ACGCAGCTCAGTAACAGTCC
*Sod2*	AGCTGCACCACAGCAAGCAC	TCCACCACCCTTAGGGCTCA
*Cat*	TCCGGGATCTTTTTAACGCCATTG	TCGAGCACGGTAGGGACAGTTCAC
*Gpx1*	CGGTTTCCCGTGCAATCAGT	ACACCGGGGACCAAATGATG
*Gsr*	TGGCACTTGCGTGAATGTTG	CGAATGTTGCATAGCCGTGG
*Nfe2l2*	GGTTGCCCACATTCCCAAAC	GGCTGGGAATATCCAGGGC
*Hmox1*	GCGAAACAAGCAGAACCCA	GCTCAGGATGAGTACCTCCCA
*Nos2*	CGAAACGCTTCACTTCCAA	TGAGCCTATATTGCTGTGGCT
*Tnf*	AGAGGCACTCCCCCAAAAGA	CGATCACCCCGAAGTTCAGT
*Il1*β	TGCCACCTTTTGACAGTGATG	TTCTTGTGACCCTGAGCGAC
*Il10*	TTGAACCACCCGGCATCTAC	CCAAGGAGTTGCTCCCGTTA
*Bcl2*	GACAGAAGATCATGCCGTCC	GGTACCAATGGCACTTCAAG
*Bax*	CTGAGCTGACCTTGGAGC	GACTCCAGCCACAAAGATG
*Casp3*	GAGCTTGGAACGGTACGCTA	CCGTACCAGAGCGAGATGAC

### Western Blotting Analyses

Protein blotting analyses were performed as previously demonstrated (Yin et al., 2019). The employed antibodies included mouse antibody to Nrf2 (MAB3925, 1:500; R & D System), HO-1 (sc-390991, 1:750; Santa Cruz Biotechnology, Santa Cruz, CA, United States), β-actin (MAB8929, 1:500; R & D System), and goat anti-mouse IgG (sc-2039, 1:5000; Santa Cruz Biotechnology, Santa Cruz, CA, United States). The proteins were visualized using an enhanced chemiluminescence detection kit (Bio-Rad, Hercules, CA, United States) following the manufacturer’s protocol. Images were analyzed using the Kodak Image Station 2000R (Eastman Kodak Company, Rochester, NY, United States). Protein bands intensity were referenced to β-actin, and the data expressed as percentage difference relative to controls.

### Histological Examination

The kidneys were immediately removed from sacrificed animals and fixed for 24 h at 27–30°C in 10% neutral formalin. The tissues were dehydrated in ascending series of alcohol, cleared in xylene, embedded in paraffin wax, and sectioned at 5-μm thick sections. The paraffin sections were rehydrated and stained with hematoxylin and eosin according to Drury and Wallington (1980). Sections were examined using a Nikon microscope (Eclipse E200-LED, Tokyo, Japan). Images were obtained at an original magnification of 400×.

### Statistical Analysis

The recorded findings were presented as the mean ± standard deviation (SD). Data for multiple variable comparisons were analyzed by one-way analysis of variance (ANOVA) followed Duncan’s test as a *post hoc* test, and a statistically significant variation was considered at *P* < 0.05.

## Results

### Pb Content in the Kidney Tissue

**FIGURE 1:**
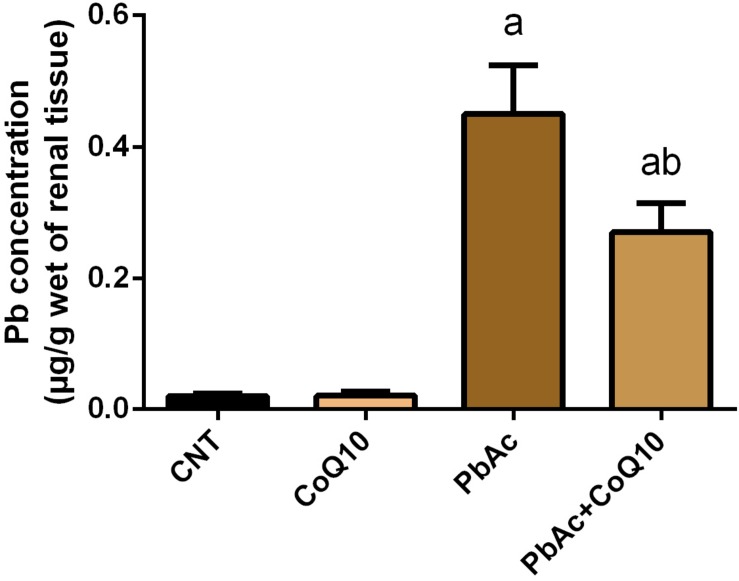
Pb content in the renal tissue following treatment with PbAc (20 mg/kg) and/or CoQ10 (10 mg/kg). Results are presented as mean ± SD (*n* = 7). ^a^ indicates significant difference vs. the control group at *P* < 0.05; ^b^ indicates significant difference vs. the PbAc treated group at *P* < 0.05.

PbAc injection (20 mg/kg) for 7 days showed a significant accumulation (*P* < 0.05) of Pb in the renal tissue as compared to its concentration in the control group. Meanwhile, the levels of Pb in CoQ10 treated rats (10 mg/kg) were very low and similar to its levels in the control group. Interestingly, the treatment with PbAc and CoQ10 decreased the level of Pb when compared to the PbAc exposed group, reflecting that CoQ10 may have a potential chelating activity or may augment the excretion of Pb from the body (Figure 1).

### Protective Role of CoQ10 in the Kidney Function Parameters Upon PbAc Exposure

**FIGURE 2:**
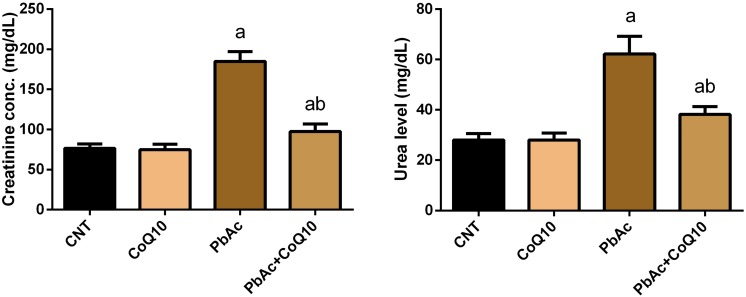
Levels of serum urea and creatinine following treatment with PbAc (20 mg/kg) and/or CoQ10 (10 mg/kg). Results are presented as mean ± SD (*n* = 7). ^a^ indicates significant difference vs. the control group at *P* < 0.05; ^b^ indicates significant difference vs. the PbAc treated group at *P* < 0.05.

PbAc injected rats showed a significant increase (*P* < 0.05) in the assessed renal function indices when compared to their levels in the control group. CoQ10 supplementation alone did not change the concentration of serum creatinine and urea. Meanwhile, CoQ10 administration 1 h after PbAc decreased the elevated renal physiological markers as compared with PbAc injected rats without CoQ10 (Figure 2); indicating its renoprotective effect.

### Protective Role of CoQ10 in Oxidative Stress Indices in the Renal Tissue Upon PbAc Exposure

**FIGURE 3:**
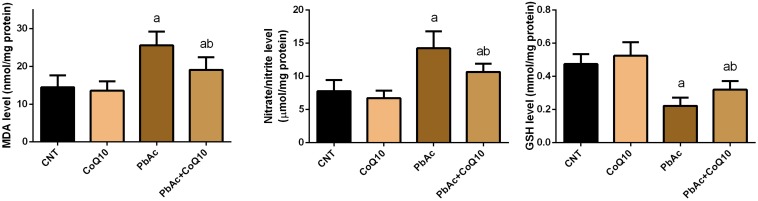
Levels of oxidative stress markers in the renal tissue following treatment with PbAc (20 mg/kg) and/or CoQ10 (10 mg/kg). Results are presented as mean ± SD (*n* = 7). ^a^ indicates the significant difference vs. the control group at *P* < 0.05; ^b^ indicates significant difference vs. the PbAc treated group at *P* < 0.05.

**FIGURE 4:**
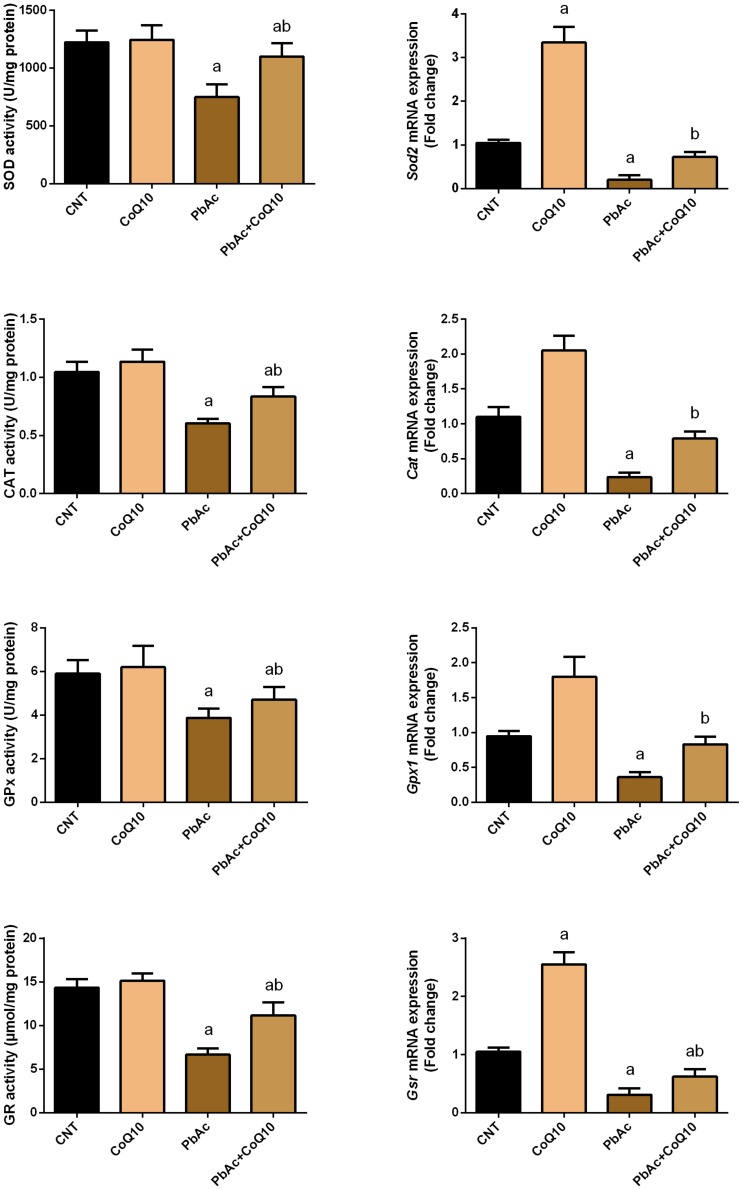
Activity and mRNA expression of the antioxidant enzymes in the renal tissue following treatment with PbAc (20 mg/kg) and/or CoQ10 (10 mg/kg). Activity of antioxidant enzymes are presented as mean ± SEM (*n* = 7). Real-time PCR results are figured as mean ± SD of triplicate assays and adjusted to *Actb*. ^a^ indicates significant difference vs. the control group at *P* < 0.05; ^b^ indicates significant difference vs. the PbAc treated group at *P* < 0.05.

Exposure to PbAc correlated with a significant increase (*P* < 0.05) in malondialdehyde, nitric oxide biosynthesis and *Nos2* mRNA expression, along with a decrease in glutathione content. Moreover, the assessed antioxidant detoxifying enzymes including SOD, CAT, GPx, and GR were deactivated. In addition, PbAc intoxication downregulated significantly (*P* < 0.05) the mRNA expression of *Sod2*, *Cat*, *Gpx1*, and *Gsr* as compared with their corresponding control values. CoQ10 treated rats showed a non-significant increase in the levels of the estimated antioxidant molecules and their mRNA expression. Expectedly, the treatment of rats with CoQ10 following injection with PbAc led to a significant reduction in oxidative stress in the renal tissue through inhibiting lipid peroxidation, nitric oxide production, and downregulation of *Nos2* expression and enhancing the activity of antioxidants and their mRNA expression (Figures 3, 4).

**FIGURE 5:**
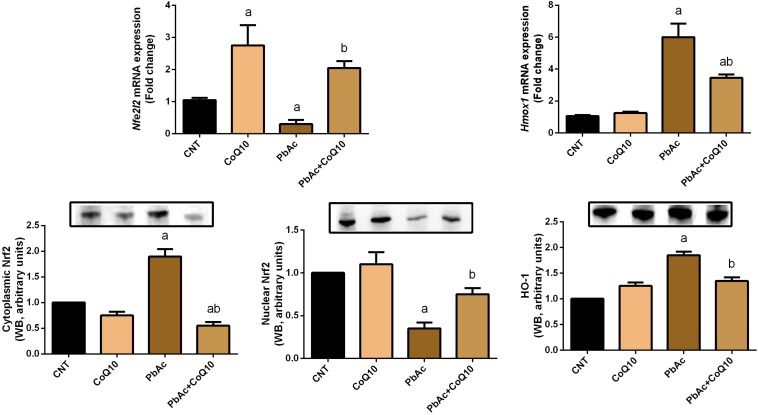
mRNA and protein expression of Nrf2 and HO-1 in the renal tissue following treatment with PbAc (20 mg/kg) and/or CoQ10 (10 mg/kg). Real-time PCR findings are presented as mean ± SD of triplicate assays and referenced to *Actb*. ^a^ indicates significant difference vs. the control group at *P* < 0.05; ^b^ indicates significant difference vs. the PbAc treated group at *P* < 0.05.

To further investigate the molecular antioxidant capacity of CoQ10 against PbAc-induced oxidative insults in the kidney tissue, the expression of *Nfe2l2* and *Hmox1* was analyzed using real-time PCR and Western blotting analyses. PbAc intoxicated rats showed a significant decrease (*P* < 0.05) in the transcriptional levels and protein expression of Nrf2 and HO-1 when compared with those in the control group. The administration of CoQ10 alone elicited a significant elevation in Nrf2 and HO-1 expression as compared with the control group. In comparison to the PbAc injected group, CoQ10 treatment along with PbAc was found to upregulate significantly the downregulated mRNA expression of *Nfe2l2* and *Hmox1*; suggesting the potent antioxidant capacity of CoQ10 against PbAc-induced oxidative stress in the renal tissue (Figure 5).

### Protective Role of CoQ10 in PbAc-Induced Inflammation in the Renal Tissue

**FIGURE 6:**
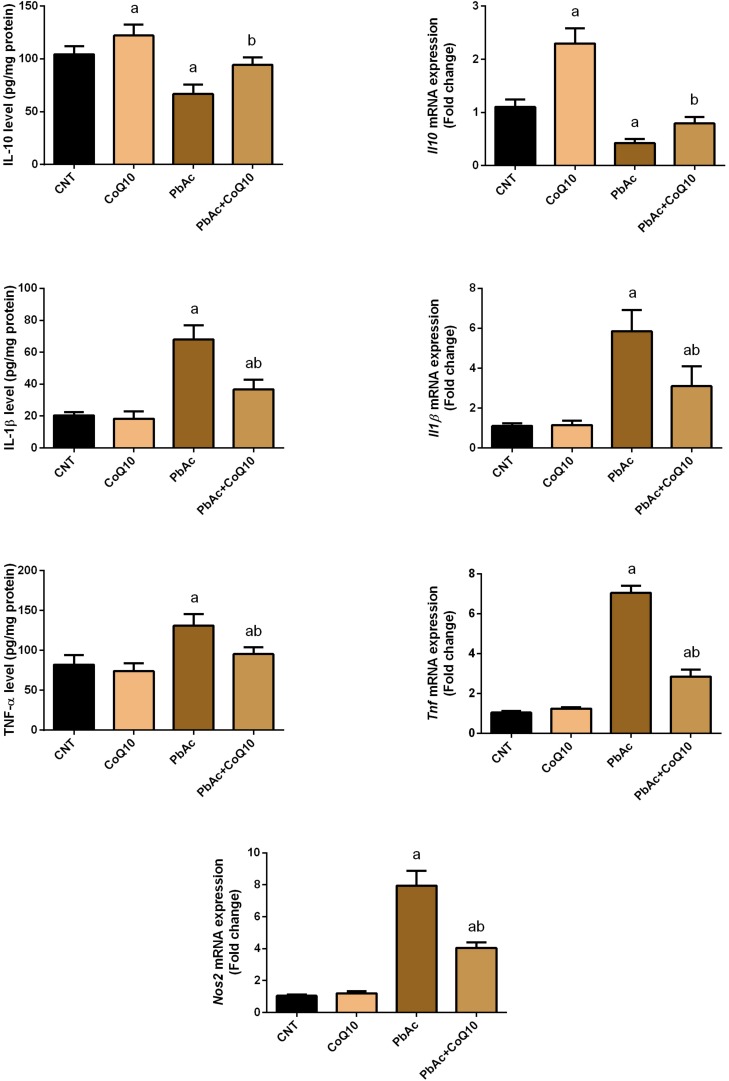
Levels and mRNA expression of tumor necrosis factor-α, interleukin-1β, interleukin-10 and inducible nitric oxide synthase mRNA expression in the renal tissue following treatment with PbAc (20 mg/kg) and/or CoQ10 (10 mg/kg). ELISA readings are presented as mean ± SD (*n* = 7). Real-time PCR results are presented as mean ± SD of triplicate assays and adjusted to *Actb*. ^a^ indicates significant difference vs. the control group at *P* < 0.05; ^b^ indicates significant difference vs. the PbAc treated group at *P* < 0.05.

The obtained findings confirmed the involvement of inflammation as an essential mechanism of toxicity following exposure to heavy metals. PbAc injection was found to initiate an inflammatory response in the renal tissue as indicated by the increased levels of pro-inflammatory cytokines (TNF-α and IL-1β) and a decrease in the protein levels of the anti-inflammatory cytokine, IL-10 as compared to their control levels. Meanwhile, no alteration in the levels of these inflammatory mediators was recorded in CoQ10-treated rats. In addition, TNF-α and IL-1β levels were decreased and IL-10 level was increased in the PbAc + CoQ10 treated group when compared to the PbAc exposed rats. In agreement with the biochemical analysis, real-time PCR results showed a significant (*P* < 0.05) upregulation in the mRNA expression of *Tnf*, *Il1b* and *Nos2* in PbAc exposed rats. However, CoQ10 downregulated *Tnf*, *Il1b* and *Nos2* gene expression and increased mRNA expression of *Il10* in response to PbAc, reflecting its anti-inflammatory activity (Figure 6).

### Protective Role of CoQ10 in PbAc-Induced Apoptosis in the Renal Tissue

**FIGURE 7:**
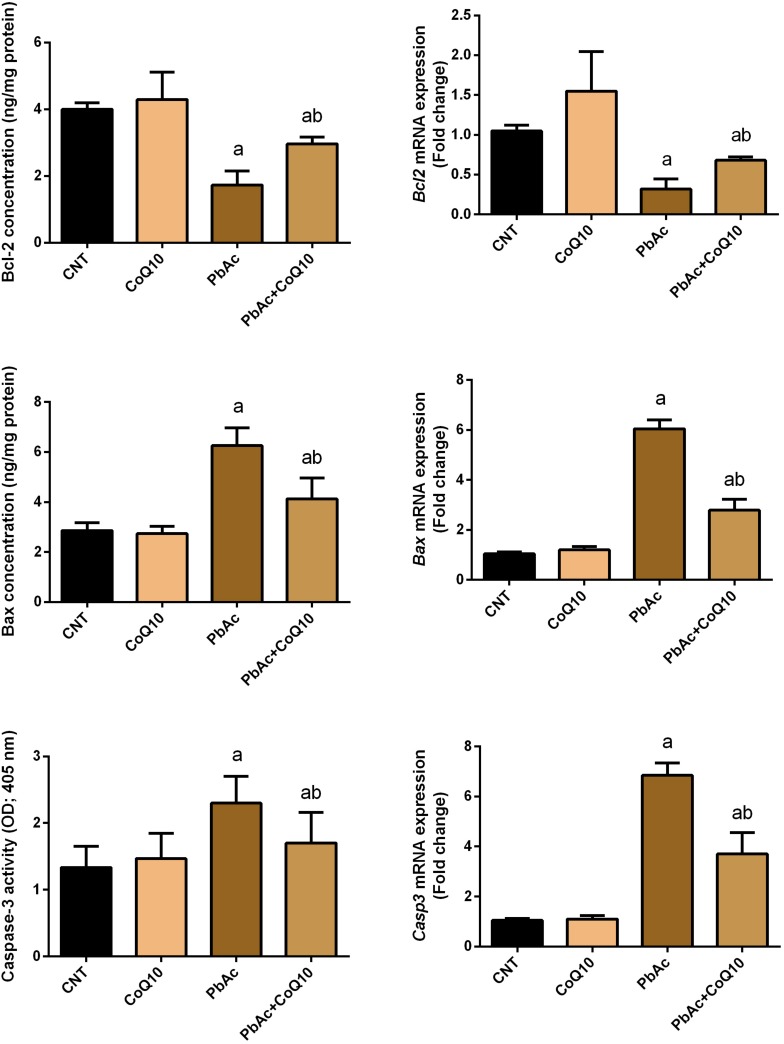
Levels and mRNA expression levels of the apoptotic proteins (Bcl-2, Bax and caspases-3) in the renal tissue following treatment with PbAc (20 mg/kg) and/or CoQ10 (10 mg/kg). ELISA readings are presented as mean ± SD (*n* = 7). Real-time PCR results are presented as mean ± SEM of triplicate assays and adjusted to *Actb*. ^a^ indicates significant difference vs. the control group at *P* < 0.05; ^b^ indicates significant difference vs. the PbAc treated group at *P* < 0.05.

To further reveal the potential nephroprotective efficacy of CoQ10 in preventing PbAc-induced renal apoptosis, we assessed the levels of Bcl-2, Bax and caspase-3, and found that PbAc exposure enhanced apoptotic events through increasing significantly (*P* < 0.05) Bax and caspase-3 protein and mRNA expression and downregulating Bcl-2 protein and mRNA expression. CoQ10 supplementation alone did not affect the protein or mRNA expression levels of these apoptotic markers. Interestingly, CoQ10 administration along with PbAc significantly prevented the apoptotic cascade caused by PbAc intoxication (Figure 7).

### Histopathological Changes in the Renal Tissue Following PbAc With or Without CoQ10 Administration

**FIGURE 8:**
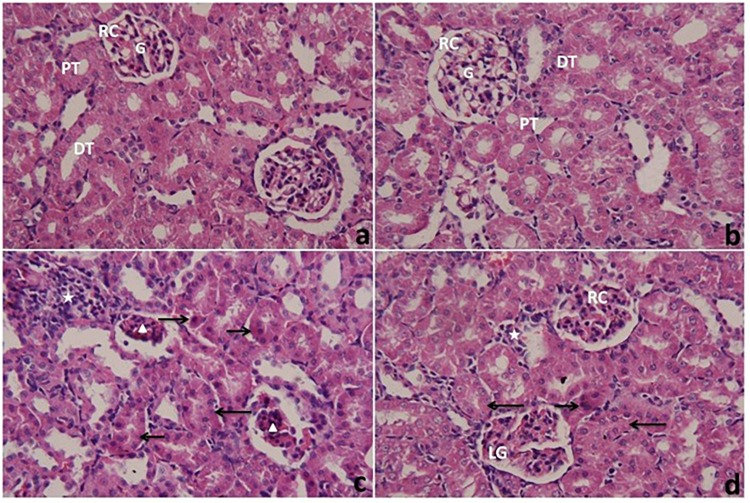
Light microscope photomicrographs of kidneys of rats treated with CoQ10 and lead acetate (PbAc) for 7 days. Cross sections of kidneys were stained with hematoxylin and eosin (400×). **(a,b)** kidneys from control and CoQ10 groups, respectively, exhibiting renal corpuscles (RC) with normal architecture of glomeruli (G) and urinary spaces. Note, normal structures of renal tubules consist of proximal tubules (PT) and distal tubules (DT). **(c)** kidney from PbAc-treated rat showing necrotic and shrunken glomeruli (arrowhead), and numerous inflammatory cells (star) in the interstitium. Most tubular cells showed pyknosis (right arrow), and are cloudy swelled (left arrow) with a small lumen of their tubules. **(d)** kidney from the post-treated group with CoQ10 against PbAc showing a glomerulus similar to normal (R) and also a lobulated glomerulus (LG). Most of the tubular cells have cloudy swelling with small or obstructed lumen (left arrow). There are few deeply eosinophilic cells representing necrotic cells (right arrow) and moderate inflammatory cells in the interstitial tissue (star).

Histopathological examination of the kidneys of the control and CoQ10-treated groups (Figures 8a,b, respectively) with light microscopy showed typical histology of renal corpuscles (consist of glomerulus, urinary space and Bowman’s capsule), the distal and proximal convoluted tubules. The kidneys of rats treated with PbAc (PbAc group) (Figure 8c) showed some pathological changes such as necrotic and shrunken glomeruli, and a frequent presence of pyknotic nuclei in the tubular epithelial. The convoluted tubules exhibited cloudy swelling and a small lumen. There was also a severe infiltration of inflammatory cells in interstitial tissues. Post-treatment of rats with CoQ10 (Figure 8d) showed a few deeply eosinophilic cells representing necrotic cells in the epithelium lining the renal tubules. The convoluted tubules exhibited cloudy swelling with a small or non-visible lumen. There were moderate inflammatory cells in the interstitium. Some glomeruli exhibited a nearly normal structure, while others were lobulated.

## Discussion

Exposure to heavy metals including Pb and its related compounds is correlated with severe adverse effects on biological systems including plants, animals and humans (Jaishankar et al., 2014). In order to identify agents that may prevent or minimize the side effects produced following exposure to heavy metals, the current study examined the possible nephroprotective effect of CoQ10 against PbAc-induced renal injury in rats. Injection of PbAc resulted in the deposition of Pb in the renal tissue. The kidneys are the main targets of heavy metal toxicity due to their ability to reabsorb and accumulate divalent ions (Lentini et al., 2017). Once absorbed, Pb is metabolized and detoxified in the liver and passes through the renal transporter system and accumulates in the kidney and other body organs (El-Khishin et al., 2015). The accumulation of Pb has been previously reported and this accumulation was coupled with deformations in kidney structures and in particular the proximal tubules (El-Khishin et al., 2015; Mabrouk, 2018). The accumulation of Pb was associated with increased serum urea and creatinine indicating the disturbance in kidney function. Increased levels of kidney function indices have been recorded previously (El-Khishin et al., 2015; Mabrouk, 2018; Shafiekhani et al., 2019). The authors suggested that this increase was due to the renal parenchymal damage and the decreased glomerular filtration rate. Antioxidants supplementation was suggested to decrease the deposition of Pb in the renal tissue through chelation and facilitated excretion (El-Khishin et al., 2015) and was found to protect the renal tissue in different experimental models (Chen X. et al., 2019; Chen Y. et al., 2019; Li et al., 2019). Here, CoQ10 administration along with PbAc decreased Pb concentration in the renal tissue. In addition, CoQ10 restored the elevated levels of serum urea and creatinine upon PbAc exposure indicating its renoprotective activity through maintaining membrane stability and integrity, and hence preventing leakage of these nitrogenous biomarkers into the circulation. Our results are in agreement with those of Ustuner et al. (2017). The authors demonstrated that CoQ10 protected the kidney tissue by decreasing the elevated serum levels of urea and creatinine in response to gentamicin-induced kidney injury in rats. In another experimental model, CoQ10 decreased also the increased kidney function indices in radiated rats, and this effect may have been due to its ability to protect the renal tissue (Ki et al., 2017).

The kidney is particularly vulnerable to Pb-induced oxidative reactions due to the relatively long residence time of Pb in the renal tissue (Bravo et al., 2007). Indeed, exposure to PbAc disturbed the redox homeostasis through increasing MDA, a lipid peroxidation product, and nitric oxide by upregulating *Nos2* mRNA expression. Additionally, PbAc decreased the content of GSH, and the antioxidant enzyme activity and mRNA expression. Moreover, Pb exposure was found to downregulate the expression of Nrf2 and HO-1 in the renal tissue. Indeed, oxidative stress has been suggested to play a crucial role in Pb-induced nephrotoxicity (Zhang et al., 2019). Pb is known to enhance ROS generation, lipid peroxidation and to deactivate antioxidant molecules and reduce the expression of the corresponding gene (Liu et al., 2010; Gargouri et al., 2019). Also, Pb competes with sulfhydryl group and trace elements including Cu, Zn, Se, and Fe in the antioxidant enzymes leading to their deactivation (Matovic et al., 2015; Mabrouk and Ben Cheikh, 2016). Nrf2 and HO-1 protect the cells against xenobiotics through activating the antioxidant and detoxifying defense system. The inhibited Nrf2 and HO-1 following PbAc poisoning may also explain the inactivation of antioxidant molecules in the current study. These findings are in line with a previous report (Liu et al., 2017). Interestingly, CoQ10 administration along with PbAc balanced the redox homeostasis in the renal tissue as indicated by the decrease in the levels of oxidants, the increase in the levels of the antioxidant molecules and the upregulation of Nrf2 and HO-1 expression. It has been shown that CoQ10 administration is able to decrease MDA and nitric oxide production along with increasing GSH content and the activity of GPx1, SOD, and CAT following doxorubicin-induced oxidative damage in rats (Mustafa et al., 2017). Moreover, CoQ10 supplementation has been found to activate Nrf2/HO-1 signaling in response to carboplatin-induced renal damage in mice (Kabel and Elkhoely, 2017). In the current study, the upregulated Nrf2 and HO-1 expression following CoQ10 supplementation may explain the increased levels of the antioxidant defense molecules in response to PbAc in the renal tissue.

There is growing interest in measuring the levels of cytokines and other inflammatory mediators and connecting changes in their expression with the clinical findings and mechanisms behind the pathophysiology of Pb toxicity. Our data indicated that exposure to PbAc led to an increase in the protein and mRNA levels of the pro-inflammatory cytokines TNF-α and IL-1β and a down-regulation in the protein and mRNA levels of the anti-inflammatory cytokine, IL-10. These results are in accordance with those of Dkhil et al. (2016), Offor et al. (2017), and BaSalamah et al. (2018) who have reported that exposure of rats to PbAc increased the levels of pro-inflammatory cytokines in the kidney tissue. The authors have also found that NF-κB expression was increased in PbAc-intoxicated rats and that NF-κB translocated from the cytosol to the nucleus where it promotes the transcription of many pro-inflammatory genes. Hence, restraining pro-inflammatory cytokine formation is a possible strategy to cure PbAc-induced nephrotoxicity. Interestingly, CoQ10 treatment prevented the robust increase in pro-inflammatory cytokines in the renal tissue of PbAc-intoxicated rats. Our results signify the anti-inflammatory effect of CoQ10. Several reports have demonstrated the anti-inflammatory effect of CoQ10. In this context, Udhaya et al. (2016) have found that CoQ10 reduces the migration of leukocytes as well as of monocytes or macrophages responsible for producing the pro-inflammatory cytokines. Furthermore, Fan et al. (2017) have reported that CoQ10 administration for a 1-week to a 4-month intervention period markedly diminished the synthesis of inflammatory cytokines. Lee et al. (2013) have reported that CoQ10 reduced the transactivation of NF-κB that may explain the decreased expression of pro-inflammatory cytokines.

To further reveal the possible nephroprotective effect of CoQ10 in blocking PbAc-induced cell loss in kidney tissue, we measured the levels of Bcl-2, Bax and caspase-3 and found that CoQ10 significantly prevented PbAc-induced up-regulation of Bax and caspase-3 protein and mRNA expression and down-regulation of Bcl-2 protein and mRNA expression. Our findings are in agreement with earlier reports. For example, Thangarajan et al. (2018) have demonstrated that PbAc intoxication in rats alters Bax/Bcl-2 expression, thereby increasing cytochrome c liberate from the mitochondrial matrix in the brain. Zhu et al. (2017) have found that PbAc decreased Bcl-2 protein and mRNA expression, and elevated Bax and caspase-3 protein and mRNA expression, thus inducing cell death in chicken brain tissues and embryonic neurocytes via the mitochondrial pathway. Furthermore, Liu et al. (2016) have found that PbAc caused a disruption in the mitochondrial integrity, the discharge of cytochrome c, the activation of caspase-3, and apoptosis in rat proximal tubular cells. CoQ10 treatment restrained the increment in Bax and caspase-3 and the decline in Bcl-2 levels in the renal tissue. In this context, Sumi et al. (2018) have demonstrated that CoQ10 prevents cytochrome c release from mitochondria and activation of caspase-3 in mouse pancreatic β-cell line and Lulli et al. (2012) have shown that CoQ10 was able to prevent apoptosis in corneal keratocytes by maintaining the mitochondrial permeability transition pore in a closed conformation. Our findings demonstrated that CoQ10 protects against PbAc-induced nephrotoxicity in rats via preventing apoptosis.

## Conclusion

**FIGURE 9:**
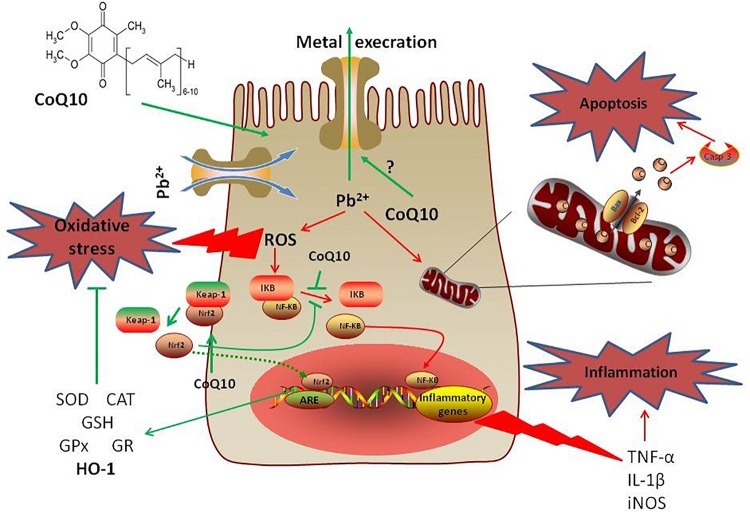
A mechanistic diagram showing the protective effect of CoQ10 against PbAc-induced renal injury. Pb^2+^ triggered ROS generation in cytoplasm mediating oxidative reactions that led to the development of inflammatory and apoptotic events. However, CoQ10 prevented the renal injury following PbAc exposure through activating the Nrf2/HO-1 pathway and inhibiting the pro-inflammatory and pro-apoptotic molecules. Green line represents stimulatory and red line represents inhibitory effects.

The present study demonstrated that intoxication with PbAc was potentially nephrotoxic and involved oxidative damage, inflammation and apoptosis. However, treatment with CoQ10 counteracted the PbAc-induced inflammation and apoptosis, and restored the antioxidative capacity, suggesting its ameliorative effect against PbAc-induced nephrotoxicity (Figure 9). Consistent with studies in mammals, CoQ10 alleviated PbAc-induced toxicity in the rats’ renal tissues via the Nrf2/HO-1 pathway.

## Data Availability Statement

The datasets generated for this study are available on request to the corresponding author.

## Ethics Statement

The animal study was reviewed and approved by the Institutional Animal Ethics Committee guidelines for animal care and use at Helwan University.

## Author Contributions

All authors listed have made a substantial, direct and intellectual contribution to the work, and approved it for publication.

## Conflict of Interest

The authors declare that the research was conducted in the absence of any commercial or financial relationships that could be construed as a potential conflict of interest.
